# Terpenic Constituents of Essential Oils with Larvicidal Activity against *Aedes Aegypti*: A QSAR and Docking Molecular Study

**DOI:** 10.3390/molecules28062454

**Published:** 2023-03-07

**Authors:** Adrián Ulises Cruz-Castillo, Luz María Rodríguez-Valdez, José Correa-Basurto, Benjamín Nogueda-Torres, Sergio Andrade-Ochoa, Guadalupe Virginia Nevárez-Moorillón

**Affiliations:** 1 Campus Coyoacán, Universidad del Valle de México, Calzada De Tlalpan No. 3016 y 3058, Ex Hacienda Coapa, Delegación Coyoacán, Ciudad de México 04910, Mexico; 2 Facultad de Ciencias Químicas, Universidad Autónoma de Chihuahua, Circuito Universitario S/N Campus Universitario II, Chihuahua 31125, Mexico; 3 Escuela Superior de Medicina, Instituto Politécnico Nacional, Plan de San Luis y Díaz Mirón S/N Col. Santo Tomas, Ciudad de México 11340, Mexico; 4 Escuela Nacional de Ciencias Biológicas, Instituto Politécnico Nacional, Prolongación de Carpio y Plan de Ayala S/N Col. Santo Tomas, Ciudad de México 11340, Mexico

**Keywords:** larvicidal activity, terpenic compounds, *Aedes aegypti*, molecular modeling

## Abstract

*Aedes aegypti* is a vector for the arbovirus responsible for yellow fever, Zika and Chikungunya virus. Essential oils and their constituents are known for their larvicidal properties and are strong candidates for mosquito control. This work aimed to develop a quantitative structure–activity study and molecular screening for the search and design of new larvicidal agents. Twenty-five monoterpenes with previously evaluated larvicidal activity were built and optimized using computational tools. QSAR models were constructed through genetic algorithms from the larvicidal activity and the calculation of theoretical descriptors for each molecule. Docking studies on acetylcholinesterase (AChE) and sterol carrier protein (SCP-2) were also carried out. Results demonstrate that the epoxide groups in the structure of terpenes hinder larvicidal activity, while lipophilicity plays an important role in enhancing biological activity. Larvicidal activity correlates with the interaction of the sterol-carrier protein. Of the 25 compounds evaluated, carvacrol showed the highest larvicidal activity with an LC_50_ of 8.8 µg/mL. The information included in this work contributes to describing the molecular, topological, and quantum mechanical properties related to the larvicidal activity of monoterpenes and their derivatives.

## 1. Introduction

Mosquitoes are responsible for more diseases than any other group of arthropods [1]. Mosquito *Aedes* (Ae.) *aegypti* acts as a vector for an arbovirus responsible for yellow fever; it is also a vector of Zika and Chikungunya virus and dengue hemorrhagic fever [2]. The World Health Organization (WHO) estimates that approximately 3.9 billion people are at risk of dengue fever; 390 million dengue infections occur annually worldwide. Of an estimated 500,000 people with severe dengue fever that require hospitalization each year, about 2.5% die due to complications [3]. Projections for 2050 have concluded that there is a potential expansion of *Ae. aegypti* and *Ae. albopictus* because of climate change implies a potential expansion of these mosquito-borne diseases [4]. In addition to these problems, cases have been identified of mosquitoes resistant to the traditionally used insecticides [5].

Throughout history, plants and insects have coexisted and evolved in parallel. Plants, in turn, have used insects as pollinators and developed a defense mechanism against insect predators [6]. In this context, essential oils and their constituents have turned out to be beneficial bioactive compounds against disease-carrying mosquitoes and other insects. [7,8]. Essential oils are substances of plant origin; they are mixtures of water-insoluble volatile secondary metabolites in different proportions, which are responsible for their biological characteristics [9]. Regarding their chemical composition, essential oils usually have phenylpropanes and terpenes, including aldehydes, alcohols, esters, and ketones. These compounds are responsible for essential oils’ fragrance and biological properties [10].

For decades, essential oils and their constituents have been used as repellents and insecticides against different species of insects [11]. For example, essential oils from plants belonging to the botanical families *Lamiaceae*, *Myrtaceae*, and *Poaceae* have been widely reported as repellent and larvicidal agents [12]. Various extracts of *Cymbopogon* have been used traditionally to repel mosquitoes [13]. This genus produces the most widely used natural repellents in the world and its activity against *Ae. aegypti* [14,15].

Reports on the pure components’ repellent and larvicidal activity are more cases than those related to essential oils. In general, the study of the pure compounds has revealed the synergistic and antagonistic effects of the components of the essential oils, demonstrating that the larvicidal activity is not only associated with the major compounds, but that other molecules present in a lesser proportion also contribute to their activity [16,17,18,19,20].

For all the above, this work aims to conduct in silico studies on the larvicidal activity of terpenes and their derivatives for the generation of predictive mathematical models that can provide insight into the design and rational search for new larvicide agents against *Ae. aegypti* and elucidate the molecular properties involved, related to its biological activity and mechanism of action.

## 2. Results

### 2.1. Quantitative Structure–Activity Relationship

**Figure 1:**
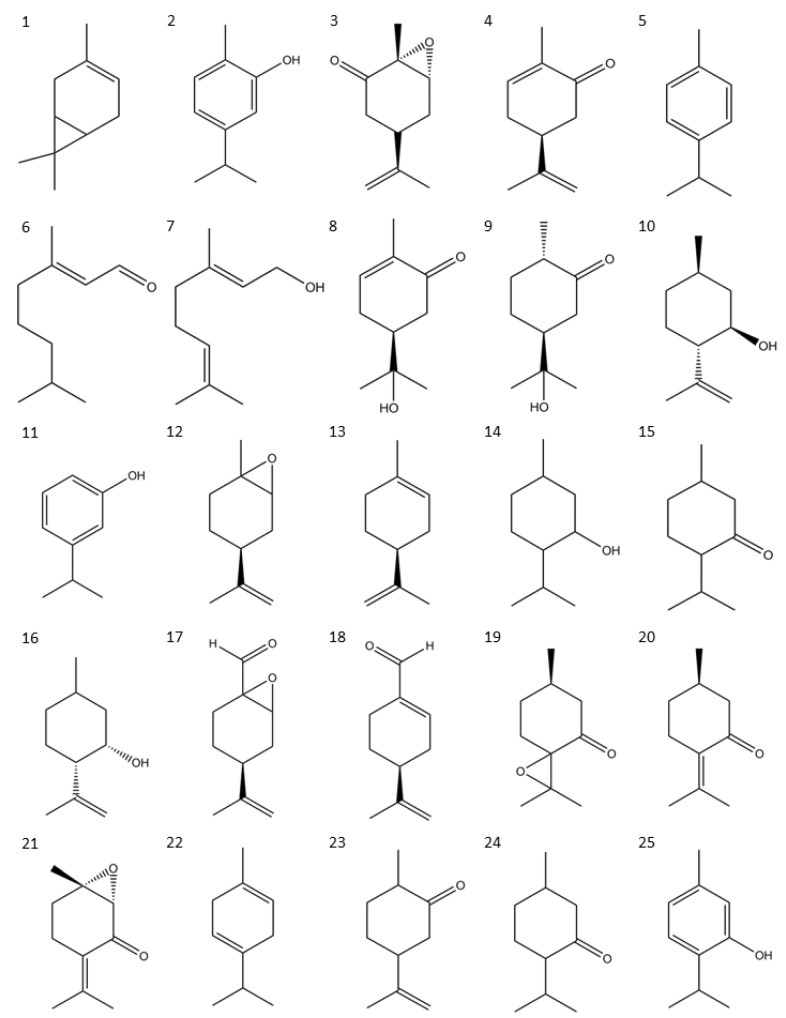
Chemical structure of terpenes and derivates evaluated. (**1**) 3-Carene, (**2**) Carvacrol, (**3**) Carvone epoxide, (**4**) Carvone, (**5**) p-Cymene, (**6**) Geranial, (**7**) Geraniol, (**8**) Hydrocarvone, (**9**) Hydrodihydrocarvone, (**10**) Isopulegol, (**11**) 3-Isopropylphenol, (**12**) Limonene epoxide, (**13**) Limonene, (**14**) Menthol, (**15**) Mentone, (**16**) Neoisopulegol, (**17**) Perillaldehyde epoxide, (**18**) Perillaldehyde, (**19**) Pulegone epoxide, (**20**) Pulegone, (**21**) Rotundifolone, (**22**) γ-Terpinene, (**23**) Trans-Dihydrocarvone, (**24**) Trans-Isopulegone, (**25**) Thymol.

The results of the larvicidal activity of terpenes and derivatives tested are included in Table 1, along with the LC_50_ values of the tested compounds. Carvacrol and thymol were the most active compounds, with LC_50_ values of 8–11 µg/mL. Although some of the compounds had a higher LC_50_ value (higher value of 1150 µg/mL), all the results were used to develop the QSAR models. The chemical structures of the tested compounds are shown in Figure 1.

**Figure 2:**
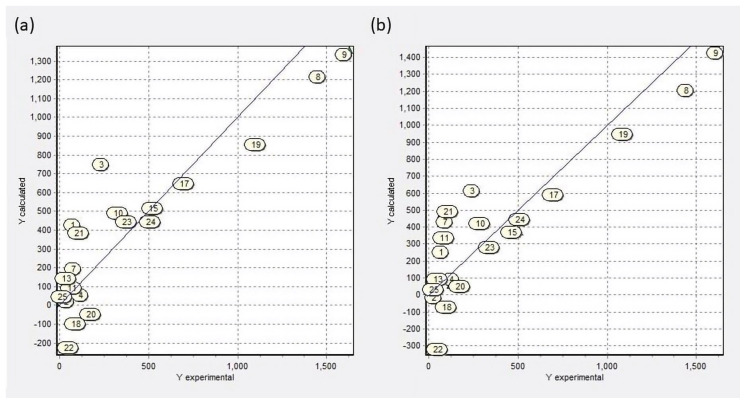
Predicted versus experimental larvicidal activity from QSAR and QPAR models. (**a**) Quantitative structure–activity relationship (QSAR) models from larvicidal activity; (**b**) quantitative property–activity relationship (QPAR) models from larvicidal activity.

Analysis of genetic algorithms demonstrates that the number of ring tertiary (nCrt) and the number of phenolic groups (nArOH) are structural descriptors related to larvicidal activity. In contrast, the number of ketones (nCO) and the number of aliphatic ethers (nROR) are descriptors inversely proportional to the biological activity. In Equation (1), the QSAR model with the most significant statistical significance value is shown. Likewise, Table 2 includes four models obtained by analysis and a plot of the predicted activity versus experimental activity for molecules using a training set for models of *Ae. aegypti*, is shown in Figure 2a, and Table 3 shows the values of the structural descriptors considered in the QSAR models by the analysis of genetic algorithms.

LC_50_ = −323.08(nCO) + 281.42(nCrt) + 1253.08(nArOH) − 361.69(nROR) − 125.67 (1)

n = 25; R^2^ = 96.07; Q^2^ = 92.06; s = 30.50; F = 85.6

Regarding molecular properties and larvicidal activity against *Ae. aegypti*, the best model included the following predictors: Ghose–Crippen octanol/water partition coefficient (ALogP), centralization (CENT), molar refractivity (AMR), and polarity number (POL). The model is expressed as shown in Equation (2):

LC_50_ = 744.70(AlogP) + 35.86(CENT) − 57.69(AMR) − 390.68(POL) − 7143.20 (2)

n = 25; R^2^ = 97.17; Q^2^ = 95.89; s = 42.918; F = 111.7

A plot of the predicted activity versus experimental activity for molecules using a training set for models of *Ae. aegypti* is shown in Figure 2b. The statistics for the other three QPAR models generated by the analysis of genetic algorithms are included in Table 2. Table 4 shows the values of the topological, molecular, and quantum-mechanic descriptors considered for the QPAR models.

### 2.2. DFT Studies

**Figure 3:**
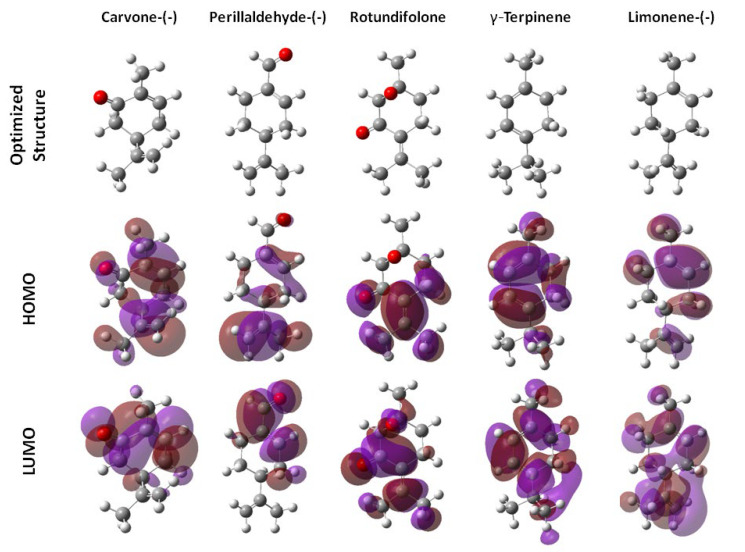
Molecular systems optimized calculated by DFT B3LYP/6-311G** level at theory and mapping of the frontier orbitals obtained by HF 6-311G**.

Chemical reactivity study shows that menthol is the monoterpene with the highest values of ionization potential (I), chemical hardness (η), and energy GAP (GAP_E_), while γ-terpinene is the chemical with the higher softness (S) and hydrocarvone the molecule with the highest LUMO energy. The quantum mechanical parameters calculated are presented in Table 5. The values are expressed in electrovolts (eV) except for the dipole moment, whose units are expressed in debyes. In Figure 3, the mapping of the frontier orbitals of the most active molecules is included.

### 2.3. Molecular Docking Studies

**Figure 4:**
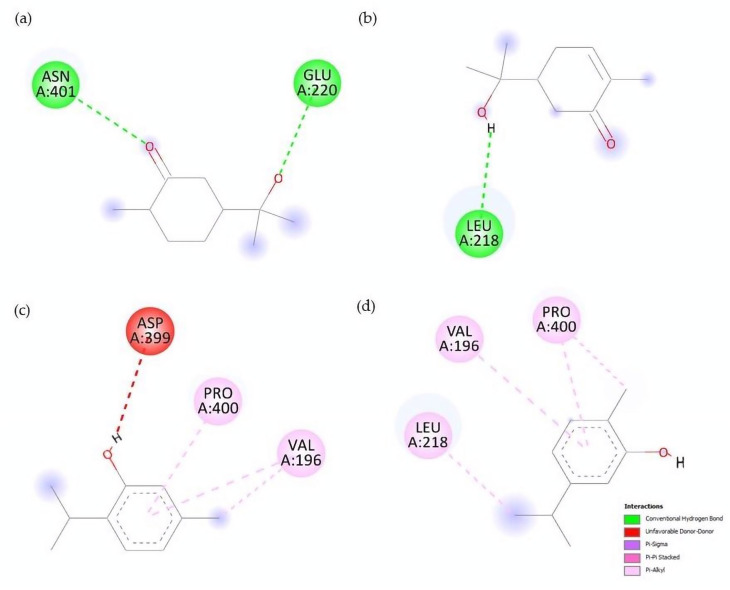
Display of AChE–ligand interactions: (**a**) hydrodihydrocarvone, (**b**) hydrodihydrocarvone, (**c**) thymol and (**d**) carvacrol.

The in silico results show that monoterpenes are better able to interact in the active site of AChE, where acetylcholine is catalyzed, in a region positioned between the V196, L218, E220, N226, V228 residues. Table 6 shows the free energy values of monoterpenes and their derivatives on the AChE and amino acids involved in the interaction. Hydrocarvone and hydrodihydrocarvone were the compounds that most efficiently bound in the AChE active site. Figure 4 shows the interactions that take place between the compounds with the highest affinity.

**Figure 5:**
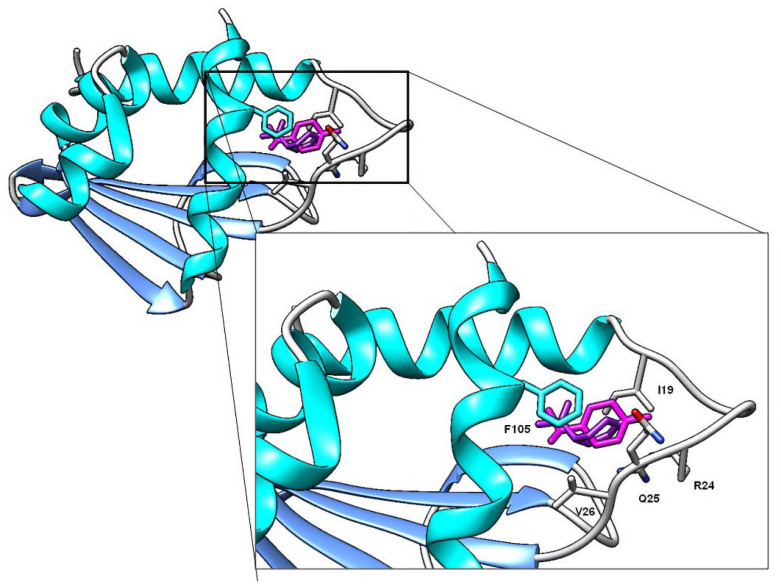
Docking studies to show the interaction of limonene (purple) and γ-terpinene (pink) with SCP-2.

**Figure 6:**
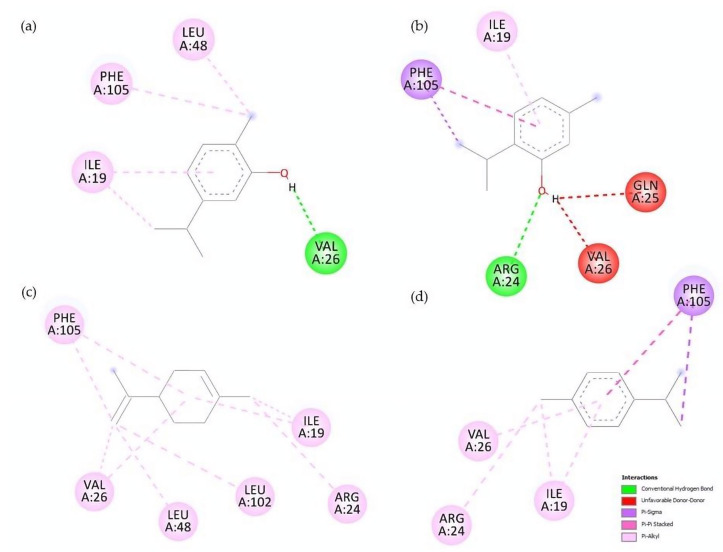
Visualization of SCP-2–ligand interactions: (**a**) carvacrol, (**b**) thymol, (**c**) limonene and (**d**) γ-terpinene.

Docking on sterol carrier protein shows that monoterpenes and their derivatives can interact in regions including I19, R24, Q25, V26, and F105 residues. Table 7 shows the binding energies (ΔG) for each terpene evaluated. Compounds are listed according to their binding energy to highlight those that had more favorable values. After the phenolic compounds, γ-Terpinene and limonene were the monoterpenes with the most efficient affinity energy, while hydrocarvone and hydrodihydrocarvone were the compounds with lower energy values. The conformation of limonene and γ-terpinene in the active site of SCP-2 can be seen in Figure 5. A visualization of the interactions that take place between the compounds with the highest affinity and the amino acid residues is observed in Figure 6.

## 3. Discussion

The development of new larvicidal agents as prospects in mosquito control is essential nowadays due to the increased incidence of diseases involving these vectors [1], the territorial expansion because of climate change [4], and the emergence of strains resistant to traditionally used insecticides [5]. Essential oils and their constituents represent an option in the search and development of new larvicidal agents due to their widely reported biological activity [7,8] and physicochemical characteristics that facilitate their extraction or synthesis [9].

Regarding the activity evaluated in vitro, it is observed that the compounds with larvicidal activities below 50 µg/mL were γ-Terpinene, limonene, thymol, and carvacrol. These last two are the most active, with LC_50_ values of 10.3 and 8.8 µg/mL, respectively. We included the in silico analysis of monoterpenes that are naturally present in essential oils and some of their derivatives to develop QSAR models, chemical reactivity, and docking molecular analysis, aiming to determine the structural and molecular characteristics that confer larvicidal activity to monoterpenes. QSAR models suggest that the ether and ketones groups hinder biological activity; the same observation was reported by Lima et al. [21], but the effect was not quantified. This is consistent with the observation that monoterpenes containing epoxy groups in their structures have less activity against *Ae. aegypti*.

QSAR model 2 (Table 2) presents nCconj descriptor as a structural element directly related to larvicidal activity, suggesting that its presence potentiates the activity; this is confirmed with the activities of 3-carene and perillaldehyde. Limonene and γ-terpinene are the terpenes with the highest larvicidal activity, but both lacks conjugated carbons or hydroxyl groups in their structure; therefore, their activity also depends on other factors. QPAR models consider the AlogP and CENT descriptors as those with a higher contribution to larvicidal activity.

Some studies have shown that the molar refractivity and hydrophilicity properties negatively correlate with coumarin toxicity against *Cx. pipens* and *Ae. aegypti* [22]. The present results reinforce the hypothesis that the descriptors associated with terpenes’ polarity (AMR, POL) contribute indirectly to their activity. On the other hand, the hydrophobic profile correlates strongly with larvicidal activity, one point that can explain the biological activity of limonene and γ-terpinene. Other studies have also shown the importance of lipophilicity properties in terpenoids and terpenes. In a previous study, models were developed based on the activity of six monoterpenes, and it was noted that when the values of the vapor pressure and lipophilicity were diminished, the lethal concentration against *Ae. aegypti* also decreased [23]. The strong participation of lipophilicity can be explained, considering that the main channel entrance of the components in the mosquito body is tactile (outer cuticle) because the larvicidal effect was mainly evaluated by immersion of larvae in an aqueous environment where the compound is applied. Thus, the molecule’s hydrophobicity plays an important role in poisoning the larvae [24].

Topological indices are numerical identities derived unambiguously from a molecular graph [25,26]. Graph center and related parameters are helpful for coding molecular structure, explaining reaction mechanisms, as well as modeling QSAR [27]. The classical definition is the minimum vertex eccentricity. This definition yielded impractically high numbers of central points [28]. We considered CENT as a topological property of importance in larvicidal activity. Active compounds like 3-carene, limonene, and γ-terpinene have small values of CENT, while the less active terpenes (pulegone epoxide, hydrocarvone and hydrodihydrocarvone) possess high values.

Information derived from the molecular orbital of the analyzed molecules can be used to derive molecular descriptors related to chemical reactivity and physical properties. The energies of the highest occupied molecular orbital (E_HOMO_) and the lowest unoccupied molecular orbital (E_LUMO_) are among the most common quantum mechanical descriptors used. Other QPAR models obtained by the analysis of genetic algorithms consider the potential of ionization (I) and chemical hardness (η) as two quantum mechanical descriptors directly related to the larvicidal activity. These two descriptors are calculated using the E_HOMO_ and E_LUMO_. The I (also known as the ionization energy) is the energy that must be provided to a system for the release of an electron, lie the strict meaning of the η corresponds to the resistance of a system to a change or deformation; it is a global property that characterizes the system as a whole, and that relates to the energy gap between the HOMO and LUMO orbitals [29]. Figure 3 shows the distribution of the HOMO and LUMO orbitals in the chemical structures of five compounds evaluated. It shows the uniform distribution of the orbitals in terpinene and limonene, the compounds with relevant larvicidal activity, while the carvone, perillaldehyde, and rotundifolone have a polar distribution. Computational tools indicate the presence of hydrophobic and hydrophilic regions on the molecular surface. However, hydrophobic interactions are more important and contribute to the increased larvicidal activity, demonstrating that aspects related to hydrophobicity are more important than steric properties to explain the biological activity.

Sesquiterpenes have also been shown to have larvicidal and repellent activity. Evidence shows that the activity of these compounds is related to vapor pressure and electronic properties as LUMO parameters [30]. This study shows in their models that the repellent activity is associated with the LUMO parameters and inversely proportional to the polarizability of the sesquiterpenes. Results of this study agree with this assertion.

Carvacrol and thymol were the compounds with the highest larvicidal activity; a similar situation was reported by our research group when evaluating their activity against *Culex quinquefasciatus* [31]. These two compounds are present in various essential oils, mainly in oregano and thyme, and their larvicidal activity has been widely reported [32,33].

Our research group has previously reported the structural importance of the phenol group in the structure and that, particularly, the position of the hydroxyl group with respect to the aliphatic chain plays an important role in biological activity. While thymol has been shown to have greater antimicrobial activity, in the case of larvicidal activity, carvacrol has been shown to be more relevant [34]. This slight increase in larvicidal activity by carvacrol has also been reported in other investigations [35]. One of these studies demonstrated that the substitution of esters, aldehydes, ethers, and acetic acid for the acidic proton of carvacrol resulted in the maintenance or reduction of larvicidal potency against *Ae. aegypti* [36].

The larvicidal activity of carvacrol and thymol may be due to different mechanisms of action. Research has suggested that these two compounds act as neurotoxic insecticides, potentiating ligand-gated chloride channels in the nervous system [37,38]. Similarly, it has been reported that thymol may work by blocking octopamine receptors [39]. On the other hand, the role of thymol and carvacrol in interacting with the cholinergic system of insects cannot be overlooked. For example, one study demonstrated the ability of carvacrol to inhibit *Aedes albopictus* acetylcholinesterase [40].

There are reports that the essential oils and their monoterpenoid components produce neurotoxic poisoning, like that produced by organophosphates and carbamates, where there is an inhibition of the acetylcholinesterase (AChE) enzyme [41,42]. Based on this proposal, we decided to use molecular docking studies on this enzyme to describe the chemical-structural properties involved in the recognition process.

In this receptor, the compounds with higher polarity were those with higher binding efficiencies, with hydrocarvone and hydrodihydrocarvone having the highest efficiency (Figure 4). However, most of the compounds exhibited higher affinity values in a region outside the acetylcholine active site, in a region between residues N226, A253, T254, V227, V228, T254, D259, H260, S273, V271. This suggests that these compounds could be AChE inhibitors allosterically, or they are not able to interact with the active site. Previous studies have shown that limonene, β-myrcene, linalool, and terpineol are potent inhibitors of AChE [43]. In silico studies have shown that linalool can interact with AChE; these results show that the linalool joins a hydrophobic site interacting with some lipophilic amino acids such as G412, G409, G412, and I413 [44]. Our results also demonstrate the importance of lipophilic residues in this region. However, some authors agree that, in most cases, there is no relationship between the inhibition of AChE and the larvicidal effects of monoterpenes [45,46]. Based on our results, we also suggest that molecular docking studies on AChE do not correlate with larvicidal activity; this could be because docking studies were performed only in one conformational structure without considering other biological properties, such as the presence of other environmental (physiological) molecules. However, docking studies were able to show the recognition properties on AChE of the target compounds that help to explain experimental data. It is also possible that the compounds exert their action on various biological targets, as has been reported in their antiprotozoal and antifungal activity.

Contrary to AChE, molecular docking studies on SCP-2 results correlate with the biological activity and QSAR models. Carvacrol and thymol, for example, are the compounds with the highest larvicidal activity and binding efficiencies. Additionally, higher-polarity compounds possess inefficient energy values; the same is observed with terpenes with epoxide groups, none of which interacted with the residue F105. Lipophilicity plays an essential role in the interaction with SCP-2; this is due to ligands with better affinity values being those that interact with L48, L102 and F105 residues (Figure 6), a question previously reported when studying the interaction of terpenes and terpenoids with SCP-2 of *Culex quinquefasciatus* [47]. In addition to carvacrol, limonene and γ-terpinene showed interactions with residues L48 and L102, generating high affinity values.

Absorbed cholesterol is redistributed within the cell, depending on physiological demands. Intracellular transporters carry out the task of mobilizing cholesterol within the cell. One lipid/sterol intracellular transport protein is the sterol carrier protein (SCP-x) [48]. The vertebrate SCP-x is a bipartite protein of a 3-ketoacyl-CoA, thiolase, and SCP-2 at the N and C-terminus [49,50]. The present study highlights the possibility that SCP-2 is also the biological target of terpenes and their derivatives, affecting the catabolism of cholesterol and branched fatty acids and bile acid intermediates [51]. However, experimental studies must be performed to elucidate this effect.

## 4. Materials and Methods

### 4.1. Larvicidal Activities

Larval mortality bioassays were performed according to the WHO recommended methodology [31,47,52] using third instar larvae of the Rockefeller lineage. To obtain the lethal concentration for 50% mortality (LC_50_) and the 95% confidence interval (CI) values, a Probit analysis was carried out with the mortality data set for each of the compounds. evaluated.

Compounds were purchased from a Sigma-Aldrich (St. Louis, MI, USA) distributor, and their chemical structures are shown in Figure 1.

### 4.2. In Silico Optimization and Descriptors Calculation

Computational analysis was carried out following the methodology previously described by our research group [47]. Each molecular system was constructed and studied by conformational analysis using Spartan 03 software (Wavefunction Inc., CA, USA) [53] and a SYBYL mechanical force field [54]. Subsequently, geometry optimization was performed at the PM3 level [55]. Once the minimum energy geometry was obtained, the descriptors were calculated using Dragon 5.4 (Talete, MI, Italy) [56].

To obtain the chemical reactivity descriptors, the Gaussian 09 (Gaussian Inc., Wallingford, CT, USA) [57] program was used through the Density Functional Theory (DFT) in the aqueous phase, using the functional B3LYP [58,59] in combination with the basic set 6-311G(d,p) in a conductive polarized continuum model (CPCM) [60]. The energy of the frontier orbitals and the Koopmans theorem [61] were applied to calculate the chemical reactivity descriptors.

### 4.3. Structure–Property–Larvicidal Activity Models

Quantitative structure–activity relationship (QSAR) and quantitative property–activity relationship (QPAR) studies were performed using the experimentally calculated CL_50_ and the theoretically calculated descriptors of each molecular system. For the construction of the models, genetic algorithms were used, which were evaluated based on four statistical variables to find the most satisfactory models [62].

The initial strategy was based on generating mathematical models exclusively using structural descriptors (QSAR) to determine the most important functional groups in larvicidal activity. Subsequently, models were generated using the physicochemical, topological, and chemical reactivity (QPAR) descriptors to find the molecular properties that stand out and relate to the LC_50_.

### 4.4. Molecular Docking Studies

Docking studies were carried out on sterol carrier protein (SCP2) reported in the RCSB Protein Data Bank (PDB ID: 1PZ4) [63] and acetylcholinesterase (AChE) of *Ae. aegypti*. The protein sequence of AChE of *Ae. aegypti* (GenBank: ABN09910.1) [64] was obtained from the database of the National Center for Biotechnology Information (NCBI). The protein was modeled through the Swiss-Model server [65,66], using as template AChE of Mus musculus (PDB ID: 2WU6) [67]. The final model was subjected to Ramachandran analysis using the Rampage server [68]. Docking analysis was done for each molecule tested experimentally on both proteins (AChE modeled and SCP2 PDB ID: 1PZ4) using the AutoDock4 software v4.2.6 (Scripps Research, CA, USA) [69]. For the docking studies, the water molecules were removed from 1PZ4, and the active site was defined considering the residues within a grid of 60 Å × 60 Å × 60 Å centered in the active site, with an initial population of 100 randomly placed individuals and a maximum number of 1.0 × 107 energy evaluations. A blind docking procedure was carried out as well. The compounds for docking were drawn in Gauss view before docking; the compounds were subjected to energy minimization using the hybrid functional B3LYP with a 6, 311G(d,p) basis set. The ΔG (Kcal/mol) values were taken from the conformation with the lowest minimum free energy of the ligand coupled on the protein targets. The figures were prepared with ChemBioOffice v 22.0 (PerkinElmer, MA. USA) [70] for the structures and Chimera 2021 (RBVI, San Francisco, CA, USA) [71] for the proteins and ligands.

## 5. Conclusions

The theoretical characterization of terpenes’ structural, molecular, and quantum mechanical properties and their derivatives are presented, as well as their relation to the larvicidal activity against *Ae. aegypti* third-instar larvae. Additionally, it is concluded that the terpenes can interact with AChE and SCP-2 and that this interaction can describe the experimental biological activity data structurally. Using tools such as QSAR and molecular docking will provide a basis for rational design and search for new larvicidal agents, taking advantage of mathematical models with statistical significance and robust tools for predicting the biological activities of terpene compounds.

## Figures and Tables

**Table 1:** LC_50_ of terpenes and derivates on *Aedes aegypti* at III instar.

Compounds		Larvicidal Activity	
No	Name	LC_50_ (µg/mL)	Chi **
1	3-Carene	49.3 (40.3–58.3)	1.223
2	Carvacrol	8.8 (7.6–10.0)	1.055
3	Carvone epoxide	217.5 (181.0–253.9)	1.331
4	Carvone	119.1 (102.1–136.0)	1.202
5	p-Cymene	86.8 (62.4–71.2)	1.642
6	Geranial	78.3 (54.9–61.7)	1.181
7	Geraniol	61.7 (40.6–42.8)	0.203
8	Lima	1470.9 (1362.13–1579.6)	3.568
9	Hydrodihydrocarvone	1628.2 (1516.9–1739.5)	4.152
10	Isopulegol	297.6 (246.0–349.2)	1.048
11	3-Isopropylpheno	60.3 (35.7–44.9)	1.969
12	Limonene epoxide	522.5 (459.2–585.8)	1.722
13	Limonene	30.5 (27.8–33.1)	0.326
14	Menthol	404.7(379.1–430.2)	1.568
15	Mentone	508.9 (482.7–535.0)	1.247
16	Neoisopulegol	554.6 (506.3–602.9)	1.051
17	Perillaldehyde epoxide	715.1 (651.4–778.8)	1.265
18	Perillaldehyde	115.8 (97.9–133.7	1.113
19	Pulegone epoxide	1116.2 (999.4–1233)	0.214
20	Pulegone	188.1 (156.9–219.1)	1.332
21	Rotundifolone	72.5 (64.4–80.6)	0.975
22	γ-Terpinene	46.5 (41.1–51.9)	0.856
23	Trans-Dihydrocarvone	361.3 (331.4–391.2)	1.323
24	Trans-Isopulegone	538.8 (506.1–571.4)	1.286
25	Thymol	10.3 (7.9–12.7)	0.0966
Tx	Temephos	0.043 (0.041–0.045)	1.668

In parenthesis, 95% confidence intervals; essential oil activity is considered significantly different when the 95% CI fails to overlap. ** Chi-square value, significant at *p* < 0.05 level.

**Table 2:** Summary of statistics of QSAR and QPAR models obtain by genetic algorithm.

	QSAR				QPAR		
Statistic	Model 2	Model 3	Model 4	Statistic	Model 2	Model 3	Model 4
n	25	25	25	n	25	25	25
R^2^	88.81	87.64	87.42	R^2^	92.87	91.4	91.28
Q^2^	85.17	84.85	84.76	Q^2^	88.57	87.44	87.29
s	33.01	32.89	32.14	s	48.01	52.77	52.91
F	82.5	82.3	81.8	F	94.1	92.8	92.8
Descriptor	Contribution			Descriptor	Contribution		
Nr07	−197.61	_WC_	_WC_	AlogP	497.5	362.2	317.04
nCconj	224.37	_WC_	_WC_	Pol	−163.49	−121.95	−121.87
nArOH	965.09	936.49	934.22	CENT	8.02	4.92	4.91
nROR	−254.81	−317.19	−379.23	ELUMO	61.38	_WC_	_WC_
nCO	_WC_	−372.97	_WC_	ƞ	_WC_	369.38	_WC_
nCrt	_WC_	258.89	242.51	AMR	_WC_	_WC_	−49.2
Intercept	136.8	142.72	168.05	Intercept	814.32	621.2	617.04

n = Number of systems evaluated, R^2^ = Square of the correlation coefficient, Q^2^ = Square of the coefficient of cross-validation, s = standard deviation, F = Fisher statistic. Nr07 = Number of 7-membered rings, nR = Ct = Number of aliphatic tertiary carbons (sp2), nCrt = Number of ring tertiary, nArOH = Number of phenolic groups, nROR = Number of aliphatic ethers, nCIR = number of circuits. AlogP = Ghose–Crippen octanol-water partition coefficient (logP), Pol = polarity number, CENT = centralization, I = Ionization potential, η = hardness chemistry, Qtot = total absolute charge, _WC_ = without contribution.

**Table 3:** Structural parameters used to derive QSAR models.

Compounds		Structural Indices							
No.	Name	Nr07	nCrt	nOH	nROR	nR = Ct	nCIR	nCO	nArOH
1	3-Carene	1	2	0	0	1	3	0	0
2	Carvacrol	0	1	0	0	0	1	0	1
3	(-)-Carvone epoxide	1	2	0	1	1	3	1	0
4	(-)-Carvone	0	1	0	0	2	1	1	0
5	p-Cymene	0	1	0	0	0	1	0	0
6	Geranial	0	1	0	0	2	0	1	0
7	Geraniol	0	1	1	0	2	0	0	0
8	Hydrocarvone	0	1	1	0	1	1	1	0
9	Hydrodihydrocarvone	0	2	1	0	0	1	1	0
10	Isopulegol	0	2	1	0	1	1	0	0
11	3-Isopropylpheno	0	1	0	0	0	1	0	1
12	(-)-Limonene epoxide	1	2	0	1	1	3	0	0
13	(+)-Limonene	0	1	0	0	2	1	0	0
14	Menthol	0	2	1	0	0	1	0	0
15	Mentone	0	2	0	0	0	1	1	0
16	Neoisopulegol	0	2	1	0	1	1	0	0
17	Perillaldehyde epoxide	1	2	0	1	1	3	0	0
18	(-)-Perillaldehyde	0	1	0	0	2	1	0	0
19	Pulegone epoxide	0	3	0	1	0	2	1	0
20	(+)-Pulegone	0	1	0	0	2	1	1	0
21	Rotundifolone	1	1	0	1	2	3	1	0
22	γ-Terpinene	0	0	0	0	2	1	0	0
23	Trans-Dihydrocarvone	0	2	0	0	1	1	1	0
24	Trans-Isopulegone	0	2	0	0	0	1	1	0
25	Thymol	0	1	0	0	0	1	0	1

Nr07 = Number of 7-membered rings, nR = Ct = number of aliphatic tertiary carbons (sp2), nCrt = number of ring tertiary, nArOH = number of phenolic groups, nROR = number of aliphatic ethers, nCIR = number of circuits, nCO = number of ketones, nOH = number of hydroxyl group.

**Table 4:** Physicochemical parameters used to derive QPAR models.

Compounds		Molecular Indices			Topological Indices		QM Indices	
No.	Name	AlogP	AMR	Qtot	Pol	CENT	I	ƞ
1	3-Carene	2.873	44.722	1.861	11	46	8.973	6.551
2	Carvacrol	3.243	46.984	2.999	14	73	8.351	6.232
3	Carvone epoxide	1.262	45.779	2.581	17	78	9.771	6.598
4	Carvone	2.361	47.174	2.495	14	73	9.308	6.053
5	p-Cymene	3.51	45.29	2.631	11	60	8.542	6.356
6	Geranial	3.19	50.199	2.566	9	102	9.05	5.701
7	Geraniol	2.934	51.182	3.367	9	102	8.859	6.792
8	Hydrocarvone	1.274	49.154	3.191	16	102	7.753	7.136
9	Hydrodihydrocarvone	1.313	48.278	3.223	16	102	10.387	7.402
10	Isopulegol	2.583	47.222	2.463	14	80	9.461	7.278
11	3-Isopropylpheno	2.757	41.943	2.12	11	80	8.523	6.254
12	Limonene epoxide	2.269	45.239	2.033	13	65	9.274	7.017
13	Limonene	3.503	46.48	1.903	11	60	8.746	6.824
14	Menthol	2.779	47.445	3.545	14	80	10.918	8.218
15	Mentone	2.597	46.52	2.575	14	80	10.712	7.066
16	Neoisopulegol	2.583	47.222	2.54	14	80	9.605	7.219
17	Perillaldehyde epoxide	1.637	45.801	2.522	16	74	9.571	6.217
18	Perillaldehyde	2.668	47.272	2.461	13	71	9.442	6.019
19	Pulegone epoxide	1.411	46.091	2.579	17	104	10.826	7.238
20	Pulegone	2.752	47.129	2.448	14	80	9.146	6.328
21	Rotundifolone	1.824	46.637	2.472	17	86	9.392	6.095
22	γ-Terpinene	3.449	47.553	1.784	11	60	7.641	5.509
23	Trans-Dihydrocarvone	2.401	46.298	2.574	14	73	9.491	6.656
24	Trans-Isopulegone	2.597	46.52	2.571	14	80	10.474	7.217
25	Thymol	3.243	46.98	3.045	14	80	8.325	6.235

AlogP = Ghose–Crippen octanol-water partition coefficient (LogP), Pol = polarity number, CENT = centralization, I = ionization potential, η = hardness chemistry, Qtot = total absolute charge.

**Table 5:** Quantum-chemical indices calculated.

Compounds		Chemical Reactivity Descriptors							
No.	Name	E_HOMO_	E_LUMO_	A	χ	µ	S	GAP_E_	m
1	3-Carene	−8.97	4.13	−4.13	−2.42	2.42	0.15	13.1	0.17
2	Carvacrol	− 8.35	4.11	− 4.11	− 2.11	2.11	0.16	12.46	1.67
3	Carvone epoxide	−9.77	3.42	−3.42	−3.17	3.17	0.15	13.19	5.59
4	Carvone	−9.3	2.79	−2.79	−3.25	3.25	0.16	12.1	3.98
5	p-Cymene	− 8.54	4.17	− 4.17	− 2.18	2.18	0.15	12.71	0.05
6	Geranial	− 9.05	2.3	− 2.34	− 3.36	3.36	0.18	11.39	4.73
7	Geraniol	− 8.85	4.72	− 4.72	− 2.06	2.06	0.14	13.58	2.4
8	Hydrocarvone	−7.75	6.51	−6.51	−0.61	0.61	0.14	14.27	3.77
9	Hydrodihydrocarvone	−10.38	4.41	−4.41	−2.98	2.98	0.13	14.8	2.52
10	Isopulegol	−9.46	5.09	−5.09	−2.18	2.18	0.13	14.55	3.75
11	3-Isopropylpheno	− 8.52	3.98	− 3.98	− 2.26	2.26	0.15	12.5	1.39
12	Limonene epoxide	−9.27	4.75	−4.75	−2.25	2.25	0.14	14.03	3.13
13	Limonene	−8.74	4.9	−4.9	−1.92	1.92	0.14	13.64	0.58
14	Menthol	−10.91	5.51	−5.51	2.7	−2.7	0.12	15.4	2.04
15	Mentone	−10.71	3.41	−3.41	−3.64	3.64	0.14	14.13	3.63
16	Neoisopulegol	−9.6	4.83	−4.83	−2.38	2.38	0.13	14.43	2.32
17	Perillaldehyde epoxide	−9.57	2.86	−2.86	−3.35	3.35	0.16	12.43	3.17
18	Perillaldehyde	−9.44	2.59	−2.59	−3.42	3.42	0.16	12.03	3.63
19	Pulegone epoxide	−10.82	3.65	−3.65	−3.58	3.58	0.13	14.47	5.68
20	Pulegone	−9.14	3.51	−3.51	−2.81	2.81	0.15	12.65	3.55
21	Rotundifolone	−9.39	2.79	−2.79	−3.29	3.29	0.16	12.19	3.74
22	γ-Terpinene	−7.64	3.37	−3.37	−2.13	2.13	0.18	11.01	0.64
23	Trans-Dihydrocarvone	−9.49	3.82	−3.82	−2.83	2.83	0.15	13.31	3.59
24	Trans-Isopulegone	−10.47	3.96	−3.96	−3.25	3.25	0.13	14.43	3.5
25	Thymol	− 8.32	4.14	− 4.14	− 2.09	2.09	0.16	12.47	1.76

E_HOMO_ = energy of the HOMO orbital, E_LUMO_ = energy LUMO orbital, A = electron affinity, χ = electronegativity μ = chemical potential, S = chemical softness, GAP_E_ = E_LUMO_ − E_HOMO_ = gap energy, m = dipole moment.

**Table 6:** Docking results on acetylcholinesterase (AChe) of *Ae. aegypti*.

No.	Molecules	Ache	Amino Acid Residues Involved in the Interaction
		ΔG (Kcal)	
9	Hydrodihydrocarvone	−6.78	E220 *, N226, V228, H260, A401 *, I413, C414
8	Hydrocarvone	−6.68	L218 *, N226, V227, V228, T254, D259
25	Thymol	−6.61	V196, N226, V228, N399, P400, C414
2	Carvacrol	−6.66	V196, L218, N226, V228, N399, I413
4	Carvone	−6.63	N226, V228, C414, Y461, L462, E485, P488, L486
20	Pulegone	−6.61	N226, V228, I413, C414 *, F457, I458, Y461, L462
25	Menthol	−6.61	L159, N226, V227, V228, A253, D259 *, H260
15	Mentone	−6.59	N226, V227, V271, V228, T254, L255, D256, H260
16	Neoisopulegol	−6.59	V227, I413, C414 *, Y461, L462, F457, I458, E485, L486, V522
10	Isopulegol	−6.58	I413, C414 *, Y461, L462, F457, I458, E485, L486, V522
23	Trans-Dihydrocarvone	−6.58	C414, Y461, L462, E485, L846
13	Limonene	−6.55	C414, Y461, L462, E485, L846, P488, V522
18	Perillaldehyde	−6.54	N226, V227, V271, V228, T254, L255, D256, H260
22	γ-Terpinene	−6.54	N226, V227, V228, I413, C414, Y461
24	Trans-Isopulegone	−6.54	I413, C414 *, Y461, L462, F457, E485, V522
21	Rotundifolone	−6.52	I413, C414 *, Y461, L462, F457,
7	Geraniol	−6.51	C414, Y461, L462, E485, P488, L486, V522
19	Pulegone epoxide	−6.51	C414, Y461, L462, E485, L846, P488, V522
17	Perillaldehyde epoxide	−6.5	N226, V227, V271, V228, T254, L255, D256, H260
6	Geranial	−6.47	C414, Y461, L462, E485, P488, L486
1	3-Carene	−6.44	L159, V228, Y258, D259, H260, V271, S273
12	Limonene epoxide	−6.4	N226, T254, L255, D256, H260, C414
3	Carvone epoxide	−6.22	C414, Y461, L462, E485, L486
5	p-Cymene	−6.12	N226, T254, L255, D256, H260, P400, C414
11	3-Isopropylphenol	−6.12	N226, A253 *, T254, D259, H260, S273, V271, P400

* Hydrogen bond interaction.

**Table 7:** Docking results on sterol carrier protein (SCP-2) of *Ae. aegypti*.

No.	Molecules	SCP-2	Amino Acid Residues Involved in the Interaction
		ΔG (Kcal)	
2	Carvacrol	−6.88	I19, N23, R24, Q25, V26 *, L48, L102, F105
25	Thymol	−6.85	I19, D20, N23, R24, Q25, V26, L48, F105
13	Limonene	−6.83	I19, R24, Q25, V26, L48, L102, F105
22	γ-Terpinene	−6.81	I19, N23, R24, Q25, V26, L48, L102, F105
14	Menthol	−6.69	I19, R24 *, Q25, V26, Q25 *, F105
4	Carvone	−6.62	R15 *, I19, D20, R24, N23, Q25, V26, F105
15	Mentone	−6.51	R15 *, I19, R24, N23, Q25, V26
20	Pulegone	−6.51	R15, I19, N23, R24 *, Q25, V26
24	Trans-Isopulegone	−6.44	R15 *, I19, D20, R24, N23, Q25, V26, L48
16	Neoisopulegol	−6.34	I19, N23, R24 *, Q25, V26, F105
21	Rotundifolone	−6.33	I19, R24 *, Q25, V26, L48, F105
10	Isopulegol	−6.26	I19, D20, R24, V26 *, L48, F105
1	3-Carene	−6.17	I19, N23, R24, Q25, V26, F105
11	3-Isopropylphenol	−6.05	D20, N23, R24, Q25, V26
12	Limonene epoxide	−6.03	I19, D20, R24, N23, Q25, V26,
23	Trans-Dihydrocarvone	−5.97	R15 *, I19, D20, R24, N23, Q25, V26 *, F105
18	Perillaldehyde	−5.95	R15, L16, I19, N23, R24 *, Q25, V26, F105
7	Geraniol	−5.88	I19, D20, N23, R24, Q25, V26, L48, L102, F105
6	Geranial	−5.76	I19, D20, N23, R24, Q25, V26
17	Perillaldehyde epoxide	−5.57	R15 *, L16, I19, N23, R24, Q25, V26
19	Pulegone epoxide	−5.51	R15 *, I19, R24, V26
5	p-Cymene	−5.32	D20, N23, R24, Q25, V26
3	Carvone epoxide	−5.23	R15, I19, D20, R24, N23, Q25, V26
8	Hydrocarvone	−5.22	I19, D20 *, R24, N23, Q25, V26, F105
9	Hydrodihydrocarvone	−5.21	R15 *, I19, D20, R24, N23, Q25, V26 *, F105

* Hydrogen bond interaction.

## Data Availability

The data presented in this study are available on request from the corresponding authors.
